# Risk score based on ten lncRNA-mRNA expression predicts the survival of stage II-III colorectal carcinoma

**DOI:** 10.1371/journal.pone.0182908

**Published:** 2017-08-10

**Authors:** Ruigang Diao, Xiaodong Mu, Tingting Wang, Shuqing Li

**Affiliations:** Yangtai Yuhuangding Hospital, Zhifu District, Yantai, Shandong Province, China; University of Texas MD Anderson Cancer Center, UNITED STATES

## Abstract

The prognosis of colorectal carcinoma (CRC) is unstable in the stage II-III patients. Patients with early stage II CRC have a relative poor prognosis while other stage III CRC patients have a better prognosis. In our work, by utilizing the expression of lncRNAs and mRNAs measured by microarray (GSE39582), we constructed a risk score staging system with Cox multivariate regression model to predict the outcome of grade II-III CRC patients. Ten genes including two lncRNAs and eight mRNAs were used to estimate the survival of stage II-III CRC patients. The patients with high risk scores have poorer survival rate those with low risk scores, significantly. These results were further validated in another three independent datasets (GSE37892, GSE33113, and GSE17536). The relationship between clinical information and were evaluated, and the risk score is independent from the other clinical information and performs better in evaluating the survival of stage II-III CRC patients. Moreover, the correlation between chemotherapy was also evaluated, and we found that both patients with or without chemotherapy have a poor survival in high risk group. Gene Set Enrichment Analysis were used to find the difference between high-risk and low-risk groups, and pathways including cell adhesion and focal adhesion were significantly enriched, suggesting that the risk score reflects the status of cell-cell physical interaction. In summary, we constructed a risk staging model for grade II-III CRC, which is independent from and performs better than clinical information.

## Introduction

Colorectal cancer (CRC) is one of the most malignant cancers worldwide, with 1.4 million new case and 693,900 deaths in 2012[1]. According to recent report in China, 376,300 new case and 191,000 death occurred because of CRC[2]. Although the prognosis of stage I and stage IV is relative stable, while a large proportion of patients relapsed early of stage II and stage III CRC. Thus, the prognosis of stage II and III CRC is important for follow up and therapy selection. However, as main prognostic method, clinical staging system failed in predicting the survival of stage II and III. Thus, the molecular biomarkers for prognosis is urgently needed.

Long non-coding RNA (lncRNA) is a kind of newly discovered RNA, with no or little protein coding ability. On the other hand, lncRNAs plays important roles in carcinogenesis and cancer development, including CRC [3–7]. For example, Increased expression of the long noncoding RNA CRNDE-h was reported to have a poor prognosis in colorectal cancer, and the level of CRDE-h is positively correlated with IRX5 mRNA expression[8]. LncRNA ENST00000430471 was found to promote proliferation, migration and inhibit the apoptosis [9]. These findings indicate that lncRNAs play important roles in CRC prognosis.

Based on previous studies, one single biomarker is not sufficient to predict the survival of CRC patients. In this vein, we used multiple genes for prognosis of CRC stage II and III patients with Cox multivariate regression model. The patients with high risk score had a significantly shorter survival time than those with low risk score, and this finding was validated in another two independent cohorts, Furthermore, the risk score is independent from the other clinical information, and performs better than the other clinical information in prognosis of stage II and III CRC patients. The risk score is effective in estimating the survival of patients underwent chemotherapy or not. Gene Set Enrichment Analysis (GSEA) showed that cell adhesion related genes were differentially expressed between high and low risk group, suggesting that the risk score reflects the cell-cell interaction status.

## Results

### Identification of survival related genes

**Fig 1:**
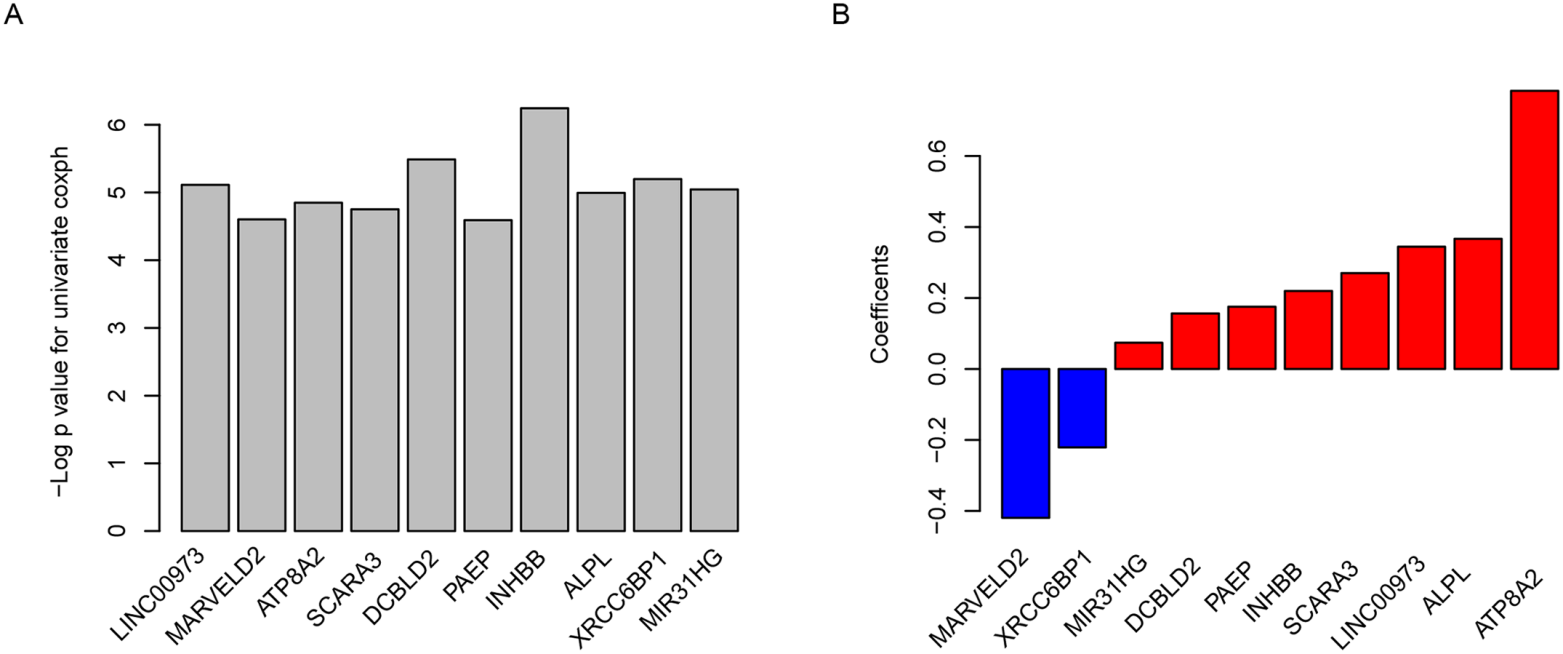
Genes selected for risk score model. The -log 10 transformed p value for Cox univariate regression (A) and multivariate coefficient (B) for each gene was shown.

We used GSE39582 microarray dataset, which consist of 481 patients with stage II and III CRC, to construct the training model. Univariate Cox regression were used to evaluate the correlation between gene expression and survival. After calculating the false discovery rate (FDR) according to the p value and false discovery rate (FDR) generated in previous step, FDR<0.05 were retained for further analysis (Fig 1a). Ten genes were obtained for further analysis, including eight mRNAs (MARVELD2, ATP8A2, SCARA3, DCBLD2, PAEP, INHBB, ALPL, XRCC6BP1) and two lncRNAs (ENSG00000240476 and MIR31HG). Then, with the ten genes, we constructed risk score with Cox multivariate model, and the coefficients of each gene were shown in Fig 1b. It is noticed that the coefficients of MARVELD2 and XRCC6BP1 are negative, indicating that these two gene have tumor-repression ability while the other eight genes are cancer genes.

### Performance of risk score in training dataset

**Fig 2:**
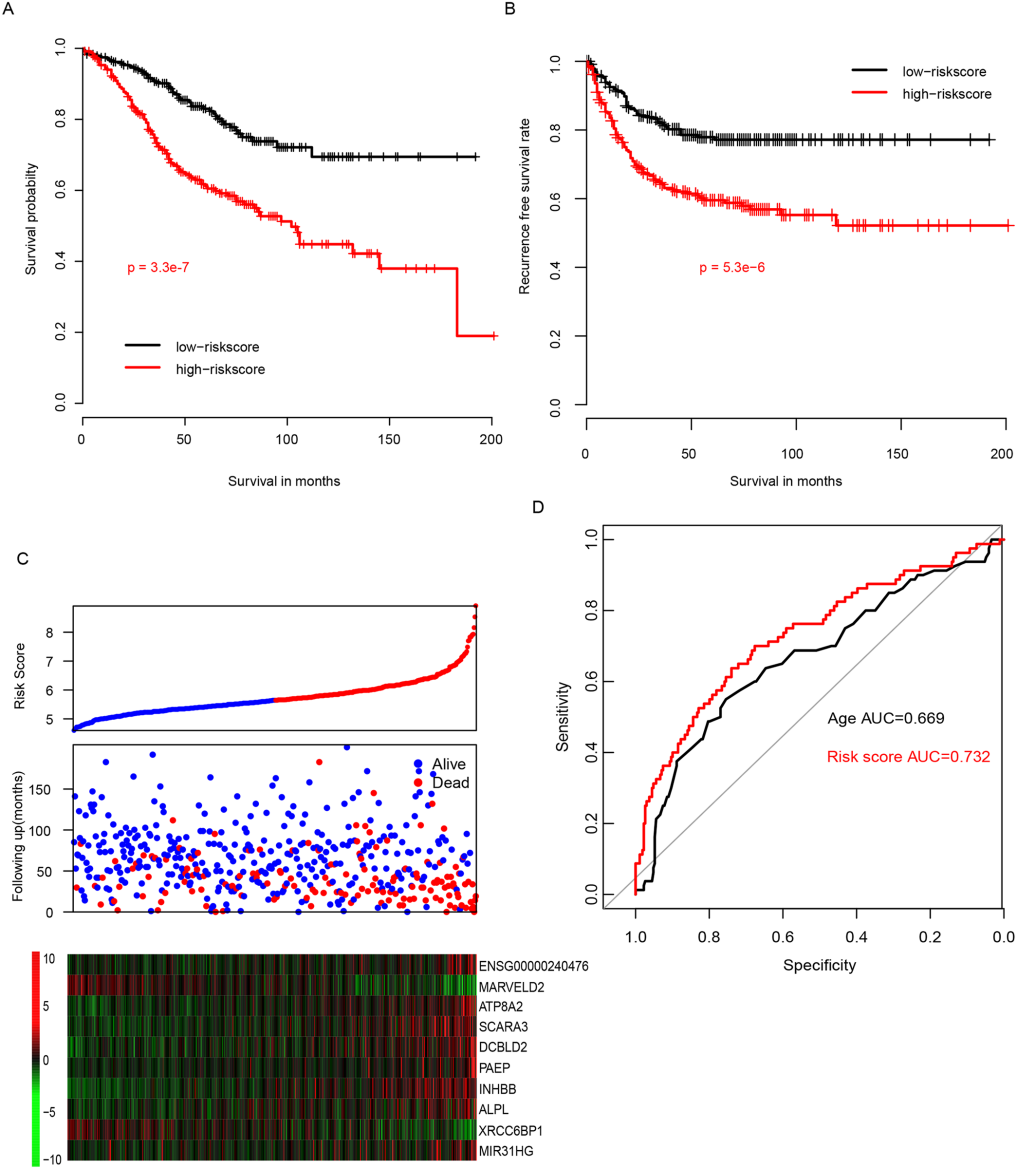
The performance of risk score on training dataset. The overall (A) and recurrence free (B) survival difference between risk-high and risk-low were shown. The detailed survival information and gene expression pattern of candidate genes were different in risk-low and risk high (C) group (Top panel, risk score, middle panel, survival status, bottom panel, candidate gene expression profile). The risk score performs better than other clinical information in predicting the 3-year survival.

To evaluate the performance of our risk score, the survival of patients with high and low risk score were evaluated, using the median risk score as cutoff. The overall survival (OS) of patients with high risk score is significantly longer than those with low risk score (Fig 2a). In addition, recurrence free survival (RFS) difference was also calculated of the high and low risk group, and the result is consistent with the OS result. As shown in Fig 2c, patients in high risk score tend to early relapse, low expression of MARVELD2/XRCC6BP1, and high expression of the other eight genes. The 3-year survival receiving operating characteristic curve was also plotted according to age, gender, stage, and risk score (Fig 2d), the area under curve (AUC) was 0.669, 0.531, 0.542, and 0.732 (age and gender not shown). We also calculated the AUC ColoGuideEx in predicting three-year survival, and AUC was 0.564, indicating that the risk score performs better in predicting the prognosis of stage II-III CRC patients than other clinical information and ColoGuideEX.

### Validation of performance of risk score in test datasets

**Fig 3:**
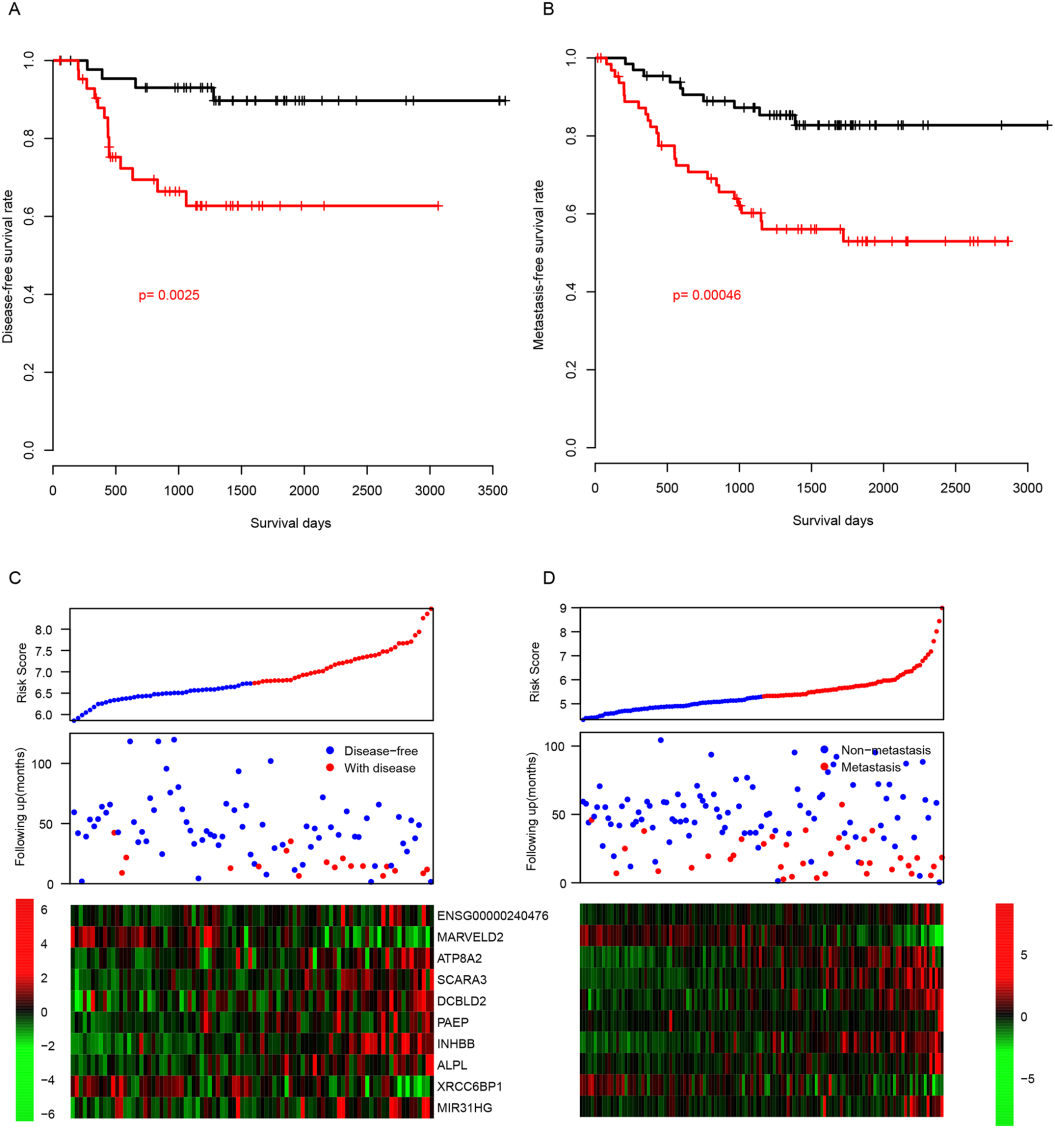
The performance of risk score in other two independent cohorts. The survival difference of risk-high and risk-low group in another two independent datasets, GSE33113 (A) and GSE37892 (B), resembles the profile of the training datasets.

After locking the coefficients of aforementioned genes, we evaluated the risk score of another two independent datasets (GSE33113, and GSE37892) and only patients in stage II and stage III were included. The disease-free survival time (DFS) in GSE33113 of low risk patients was significantly longer than these with high risk score (Fig 3a, p = 0.0025). In consistent with this, Metastasis-free survival rate of patients in GSE37892 in low risk group was significantly longer than those high risk (Fig 3b, p = 0.00046). In consistent with the observation in training datasets, in the high-risk group, early disease recurrence was reproducible in GSE33113, and early metastasis were observed in GSE37892 (Fig 3c and 3d). In addition, the expression profile of the ten genes used for risk score evaluation was similar, in compared to the training datasets. These results suggest that the risk was robust in predicting the survival of stage II and stage III CRC patients.

### Risk score, other clinical information, and chemotherapy

**Fig 4:**
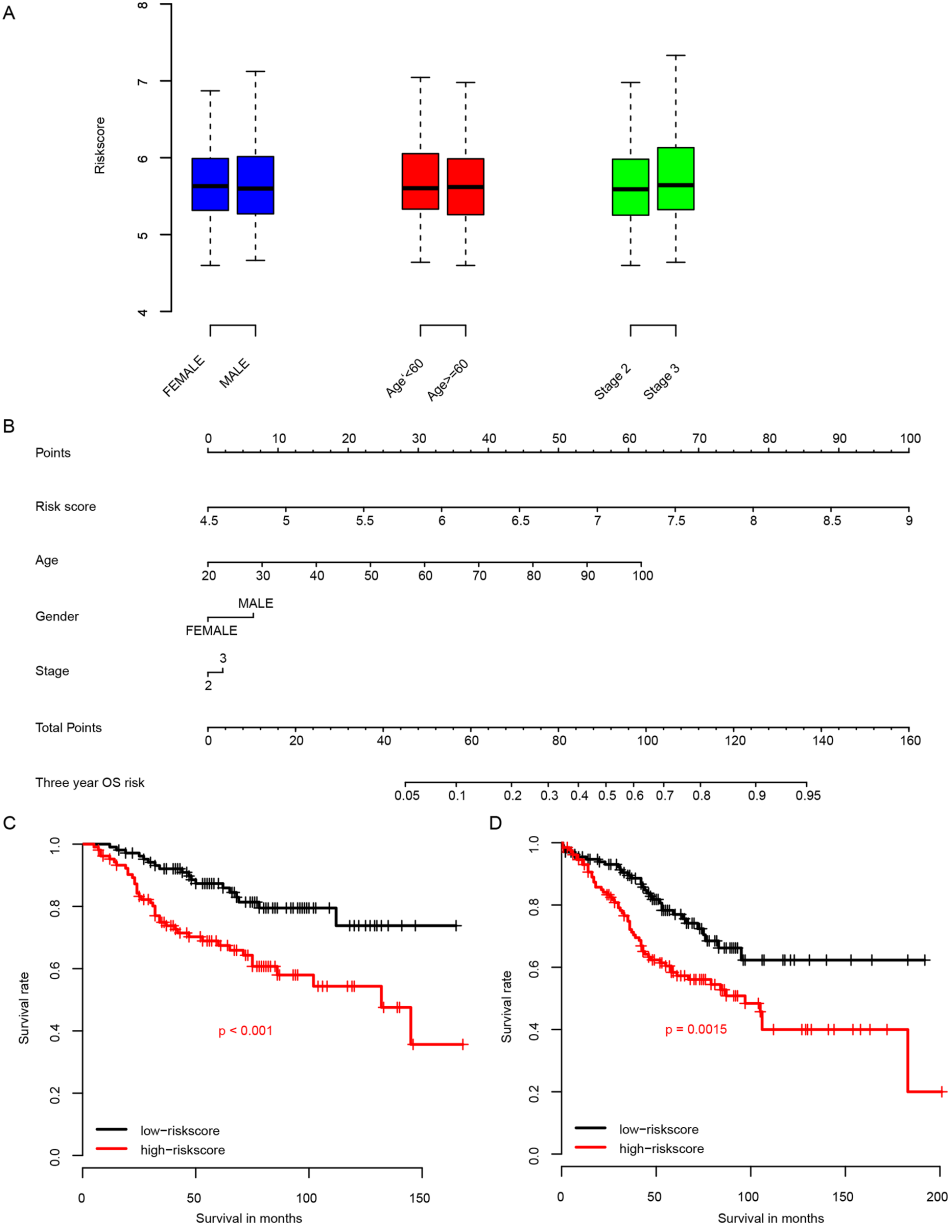
The correlation between risk score and other clinical information and its performance in predicting survival time. The risk score is independent of other clinical information (A), and contributed more in predicting 3-year survival (B, 3-year survival nomogram). Risk score is effective for patients underwent chemotherapy or not.

In order to measure the correlation between clinical information and risk score, we compared the risk score with clinical levels (Fig 4a) in the GSE39582. The risk score is not significantly different between clinical levels, including age (60 as cutoff), gender, and stage (II and III). A nomogram was drawn considering age, gender, stage, and risk score to predict the 3-year incidence possibility (Fig 4b). The contribution of gender and stage is limited, while age and risk score contributed much more, these results indicate that the risk score performs better than other clinical information in predicting the survival of stage II and stage III CRC patients.

Chemotherapy is one of the most common therapy method for CRC. In order to evaluate the correlation between chemotherapy and the risk score, we divided the patients (not) underwent chemotherapy into high risk group and low risk group using median risk score as cutoff. The overall survival rate of patients with chemotherapy (Fig 4c) and high risk score had a significantly shorter survival rate than these with low risk score. And the situation also occurred in the patients without chemotherapy (Fig 4d). These results indicate that the prognostic effect of risk score is robust and not influenced by chemotherapy.

### Altered pathways in the high risk score patients

**Fig 5:**
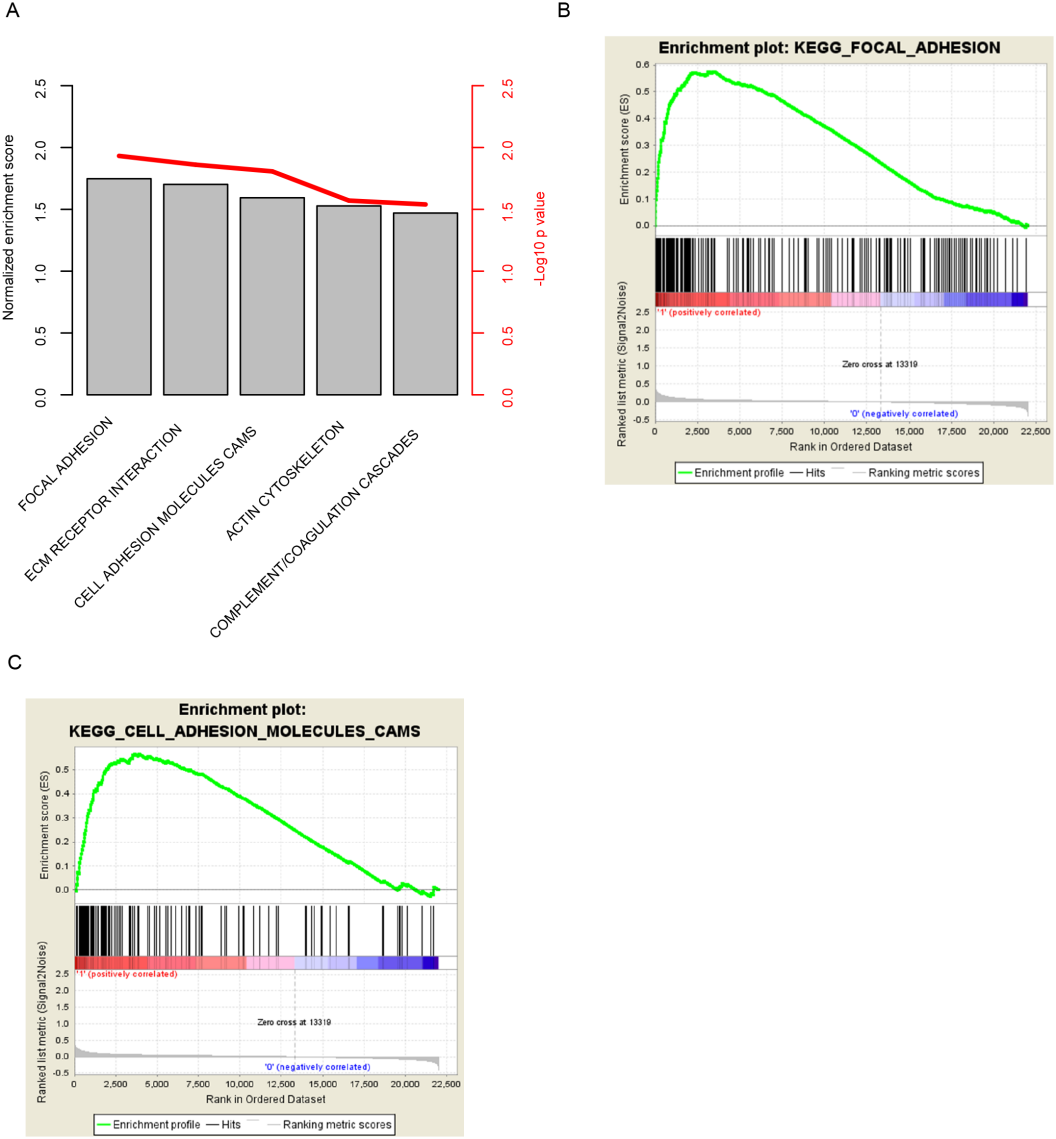
The significantly altered KEGG pathways in the risk-high group. Among these pathways (A), focal adhesion (B) and cell adhesion molecular cams (C) was noted.

The significantly altered pathways of high risk score group in contrast to the low risk group were assessed with Gene Set Enrichment Analysis (GSEA). The significantly altered pathways of high risk group include “focal adhesion”, “ECM receptor interaction”, “cell adhesion molecular cams”, and “actin cytoskeleton” (Fig 5a). We noted KEGG pathways “focal adhesion” (Fig 5b) and “cell adhesion molecular cams” (Fig 5c) associated genes were significantly enriched, suggesting that cell-cell interaction related genes of high risk group were significantly altered, thus reflecting the high viability of these samples.

## Discussion

The prognosis of stage II and III CRC patients is currently difficult by using clinical information, including TNM staging, age, etc. Thus, the molecular subtyping for prognosis is critically needed for treatment determination. Although a lot of single molecular marker for stage II/III prognosis has been reported, the clinical performance across datasets is not very satisfactory. In current report, using Cox multivariate regression on GEO datasets, we report that ten lncRNA and mRNA based risk score successfully predict the survival of stage II/III CRC patients, and this finding is validated in another two independent datasets. Compared to other clinical information, the risk score contributed more, and performs better. In addition, the risk score is also effective for patients underwent chemotherapy or not, suggesting the robustness of our risk score.

Of these ten genes, we noted two genes, the coefficient of MARVELD2 and XRCC6BP1 are negative. In consistent with this, MARVELD2 a membrane protein associated with tight junction between cells [10], and it was reported to be a prognostic gene in hepatocellular carcinoma (HCC) and cholangiocarcinomas [11]. While little is know about XRCC6BP1, except that it is a subunit of ATPase [12]. Among the cancer genes, lncRNA MIR31HG was reported to be associated with proliferation and migration in breast cancer [13]. It is also reported to regulate p16 (25908244), and controls myoblast differentiation [14]. Other genes, SCARA3 was reported to be associated with poor prognosis in breast cancer, multiple myeloma, and prostate cancer [15–17]. PAEP was reported to be a new biomarker with immunomodulatory functions in non-small cell lung cancer [18], indicating the reliability of the candidate genes for risk score evaluation.

The significantly altered pathways include focal adhesion, and other related KEGG pathways, suggesting that the risk score reflected the cell-cell interaction status of CRCs.

## Material and methods

### Data preprocessing and LncRNA matching

Raw data of GSE39582, GSE37892, and GSE33113 was downloaded in CEL format from GEO, then background correction, normalization with Robust Multiarray Averaging (RMA) were used on these data.

We downloaded the target sequences of probes (HG U133 plus 2) from Affymetrix official website (http://www.affymetrix.com/), and mapped the target sequence to the lncRNA sequences downloaded from ENSEMBL (http://asia.ensembl.org/index.html) with bowtie1 software (http://bowtie-bio.sourceforge.net/index.shtml) [19]. This mapping step allows no mismatch between Affymetrix probe target sequences and lncRNA reference sequences, and the only probes matched to the forward sequences (parameter -norc) were used for further analysis (strand-specific). Probes matched more than one lncRNAs were discarded. For lncRNAs matched multiple probes, the mean expression value was calculated and took as the expression level of corresponding lncRNAs. At last, 3505 lncRNA were used for further analysis.

For mRNAs, the gene symbols (HUGO gene symbol) provided by the HG U133 plus 2 annotation file and probes in datasets were matched, probes mapped to lncRNAs in the previous step were not used in this step.

### Prediction gene selection and cox multivariate regression model

Cox univariate regression were performed in GSE39582 dataset. P value were calculated for each gene and corrected to false discovery rate (FDR) with method “BH”. LncRNAs and mRNAs with FDR<0.05 were retained for further analysis. Cox multivariate regression were performed to estimate the risk score with the ten genes obtained in previous step.

### Statistical analysis

All statistical analysis was performed on R software (https://www.r-project.org/, v3.0.1) and R packages. Normalization and background correction of affymetrix raw data were implemented with R package “affy”. The survival analysis and cox probability hazard model was performed with R package “survival”. The ROC curve were drawn with R package “pROC” [20], and the nomogram were drawn with R package “rms”. The Gene Set Enrichment Analysis was performed with java software GSEA [21] (http://software.broadinstitute.org/gsea/index.jsp).

## Supporting information

S1 Table The hazard ratio, p value, and confidence interval of hazard ratio. (DOCX)

S2 Table AIC and BIC of clinical information and risk score. (DOCX)

S1 Fig Survival difference between high-low risk group of GSE17536 dataset. (DOCX)

## References

[1] Torre LA, Bray F, Siegel RL, Ferlay J, Lortet-Tieulent J, Jemal A. Global cancer statistics, 2012. CA: a cancer journal for clinicians. 2015;65(2):87–108. Epub 2015/02/06. doi: 10.3322/caac.21262 . 25651787 10.3322/caac.21262

[2] Siegel R, Miller K, Jemal A. Cancer statistics, 2015. CA: a cancer journal for clinicians. 2015;65(1):5–29. 25559415 10.3322/caac.21254

[3] Saus E, Brunet-Vega A, Iraola-Guzman S, Pegueroles C, Gabaldon T, Pericay C. Long Non-Coding RNAs As Potential Novel Prognostic Biomarkers in Colorectal Cancer. Frontiers in genetics. 2016;7:54 Epub 2016/05/06. doi: 10.3389/fgene.2016.00054 ; 27148353 10.3389/fgene.2016.00054 PMC4828582

[4] Li Y, Huang S, Li Y, Zhang W, He K, Zhao M, et al Decreased expression of LncRNA SLC25A25-AS1 promotes proliferation, chemoresistance, and EMT in colorectal cancer cells. Tumour biology: the journal of the International Society for Oncodevelopmental Biology and Medicine. 2016;37(10):14205–15. Epub 2016/08/25. doi: 10.1007/s13277-016-5254-0 . 27553025 10.1007/s13277-016-5254-0

[5] Lian Y, Ding J, Zhang Z, Shi Y, Zhu Y, Li J, et al The long noncoding RNA HOXA transcript at the distal tip promotes colorectal cancer growth partially via silencing of p21 expression. Tumour biology: the journal of the International Society for Oncodevelopmental Biology and Medicine. 2016;37(6):7431–40. Epub 2015/12/19. doi: 10.1007/s13277-015-4617-2 . 26678886 10.1007/s13277-015-4617-2

[6] Yang L, Qiu M, Xu Y, Wang J, Zheng Y, Li M, et al Upregulation of long non-coding RNA PRNCR1 in colorectal cancer promotes cell proliferation and cell cycle progression. Oncology reports. 2016;35(1):318–24. Epub 2015/11/05. doi: 10.3892/or.2015.4364 . 26530130 10.3892/or.2015.4364

[7] Li X, Cao Y, Gong X, Li H. Long noncoding RNAs in head and neck cancer. Oncotarget. 2016 Epub 2016/11/02. doi: 10.18632/oncotarget.12960 . 27802187 10.18632/oncotarget.12960 PMC5354695

[8] Liu T, Zhang X, Yang YM, Du LT, Wang CX. Increased expression of the long noncoding RNA CRNDE-h indicates a poor prognosis in colorectal cancer, and is positively correlated with IRX5 mRNA expression. OncoTargets and therapy. 2016;9:1437–48. Epub 2016/04/05. doi: 10.2147/OTT.S98268 ; 27042112 10.2147/OTT.S98268 PMC4795576

[9] Yang P, Xu ZP, Chen T, He ZY. Long noncoding RNA expression profile analysis of colorectal cancer and metastatic lymph node based on microarray data. OncoTargets and therapy. 2016;9:2465–78. Epub 2016/05/25. doi: 10.2147/OTT.S102348 ; 27217770 10.2147/OTT.S102348 PMC4853163

[10] Takasawa A, Kojima T, Ninomiya T, Tsujiwaki M, Murata M, Tanaka S, et al Behavior of tricellulin during destruction and formation of tight junctions under various extracellular calcium conditions. Cell and tissue research. 2013;351(1):73–84. Epub 2012/10/18. doi: 10.1007/s00441-012-1512-7 ; 23073616 10.1007/s00441-012-1512-7 PMC3536962

[11] Somoracz A, Korompay A, Torzsok P, Patonai A, Erdelyi-Belle B, Lotz G, et al Tricellulin expression and its prognostic significance in primary liver carcinomas. Pathology oncology research: POR. 2014;20(4):755–64. Epub 2014/03/22. doi: 10.1007/s12253-014-9758-x . 24652413 10.1007/s12253-014-9758-x

[12] Zeng X, Neupert W, Tzagoloff A. The metalloprotease encoded by ATP23 has a dual function in processing and assembly of subunit 6 of mitochondrial ATPase. Molecular biology of the cell. 2007;18(2):617–26. Epub 2006/12/01. doi: 10.1091/mbc.E06-09-0801 ; 17135290 10.1091/mbc.E06-09-0801 PMC1783785

[13] Shi Y, Lu J, Zhou J, Tan X, He Y, Ding J, et al Long non-coding RNA Loc554202 regulates proliferation and migration in breast cancer cells. Biochemical and biophysical research communications. 2014;446(2):448–53. Epub 2014/03/19. doi: 10.1016/j.bbrc.2014.02.144 . 24631686 10.1016/j.bbrc.2014.02.144

[14] Ballarino M, Cazzella V, D'Andrea D, Grassi L, Bisceglie L, Cipriano A, et al Novel long noncoding RNAs (lncRNAs) in myogenesis: a miR-31 overlapping lncRNA transcript controls myoblast differentiation. Molecular and cellular biology. 2015;35(4):728–36. Epub 2014/12/17. doi: 10.1128/MCB.01394-14 ; 25512605 10.1128/MCB.01394-14 PMC4301723

[15] Brown CO, Schibler J, Fitzgerald MP, Singh N, Salem K, Zhan F, et al Scavenger receptor class A member 3 (SCARA3) in disease progression and therapy resistance in multiple myeloma. Leukemia research. 2013;37(8):963–9. Epub 2013/03/30. doi: 10.1016/j.leukres.2013.03.004 ; 23537707 10.1016/j.leukres.2013.03.004 PMC3700682

[16] Bock AJ, Nymoen DA, Brenne K, Kaern J, Davidson B. SCARA3 mRNA is overexpressed in ovarian carcinoma compared with breast carcinoma effusions. Human pathology. 2012;43(5):669–74. Epub 2011/08/23. doi: 10.1016/j.humpath.2011.06.003 . 21855113 10.1016/j.humpath.2011.06.003

[17] Yu G, Tseng GC, Yu YP, Gavel T, Nelson J, Wells A, et al CSR1 suppresses tumor growth and metastasis of prostate cancer. The American journal of pathology. 2006;168(2):597–607. Epub 2006/01/27. doi: 10.2353/ajpath.2006.050620 ; 16436673 10.2353/ajpath.2006.050620 PMC1606498

[18] Schneider MA, Granzow M, Warth A, Schnabel PA, Thomas M, Herth FJ, et al Glycodelin: A New Biomarker with Immunomodulatory Functions in Non-Small Cell Lung Cancer. Clinical cancer research: an official journal of the American Association for Cancer Research. 2015;21(15):3529–40. Epub 2015/04/23. doi: 10.1158/1078-0432.ccr-14-2464 . 25901080 10.1158/1078-0432.CCR-14-2464

[19] Langmead B, Trapnell C, Pop M, Salzberg SL. Ultrafast and memory-efficient alignment of short DNA sequences to the human genome. Genome biology. 2009;10(3):R25 Epub 2009/03/06. doi: 10.1186/gb-2009-10-3-r25 ; 19261174 10.1186/gb-2009-10-3-r25 PMC2690996

[20] Robin X, Turck N, Hainard A, Tiberti N, Lisacek F, Sanchez JC, et al pROC: an open-source package for R and S+ to analyze and compare ROC curves. BMC bioinformatics. 2011;12:77 Epub 2011/03/19. doi: 10.1186/1471-2105-12-77 ; 21414208 10.1186/1471-2105-12-77 PMC3068975

[21] Subramanian A, Tamayo P, Mootha VK, Mukherjee S, Ebert BL, Gillette MA, et al Gene set enrichment analysis: a knowledge-based approach for interpreting genome-wide expression profiles. Proceedings of the National Academy of Sciences of the United States of America. 2005;102(43):15545–50. Epub 2005/10/04. doi: 10.1073/pnas.0506580102 ; 16199517 10.1073/pnas.0506580102 PMC1239896

