# Correction: Risk score based on ten lncRNA-mRNA expression predicts the survival of stage II-III colorectal carcinoma

**DOI:** 10.1371/journal.pone.0201993

**Published:** 2018-08-02

**Authors:** Ruigang Diao, Xiaodong Mu, Tingting Wang, Shuqing Li

The first two authors, Ruigang Diao and Xiaodong Mu, should be noted as contributing equally to this work.
