# Supplementary material for: Risk score based on ten lncRNA-mRNA expression predicts the survival of stage II-III colorectal carcinoma
Source: PLoS One. 2017 Aug 10;12(8):e0182908. doi: 10.1371/journal.pone.0182908 (PMC5552098; doi:10.1371/journal.pone.0182908)
Supplement: S1 Table — (DOCX) [file pone.0182908.s001.docx]

Supplementary Table 1

The hazard ratio, p value, and confidence interval of hazard ratio

| Genes | HR | pvalue | 95% CI |
| --- | --- | --- | --- |
| MIR31HG | 1.62793 | 9.01E-06 | 1.313-2.019 |
| XRCC6BP1 | 0.322991 | 6.33E-06 | 0.198-0.528 |
| ALPL | 4.332892 | 1.01E-05 | 2.260-8.308 |
| INHBB | 1.465464 | 5.70E-07 | 1.262-1.702 |
| PAEP | 1.540441 | 2.56E-05 | 1.260-1.884 |
| DCBLD2 | 2.052965 | 3.25E-06 | 1.516-2.779 |
| SCARA3 | 2.312308 | 1.77E-05 | 1.577-3.391 |
| ATP8A2 | 2.713079 | 1.42E-05 | 1.729-4.257 |
| MARVELD2 | 0.493514 | 2.49E-05 | 0.355-0.685 |
| LINC00973 | 3.075935 | 7.71E-06 | 1.880-5.033 |
