# Supplementary material for: Risk score based on ten lncRNA-mRNA expression predicts the survival of stage II-III colorectal carcinoma
Source: PLoS One. 2017 Aug 10;12(8):e0182908. doi: 10.1371/journal.pone.0182908 (PMC5552098; doi:10.1371/journal.pone.0182908)
Supplement: S2 Table — (DOCX) [file pone.0182908.s002.docx]

| Supplementary Table 2 |  |  |  |
| --- | --- | --- | --- |

| Variables | AIC | BIC |
| --- | --- | --- |
| riskscore | 334.4816 | 346.0122483 |
| Gender | 388.9203 | 400.4508985 |
| Age | 370.7171 | 382.2477105 |
| Stage | 387.8528 | 399.3834064 |
| Chemotherapy | 388.3221 | 399.8527511 |
