# Supplementary material for: Risk score based on ten lncRNA-mRNA expression predicts the survival of stage II-III colorectal carcinoma
Source: PLoS One. 2017 Aug 10;12(8):e0182908. doi: 10.1371/journal.pone.0182908 (PMC5552098; doi:10.1371/journal.pone.0182908)

Fig. 1 Survival difference between high-low risk group of GSE17536 dataset. The x-asis indicates survival month and y-axis indicates the overall survival rate.
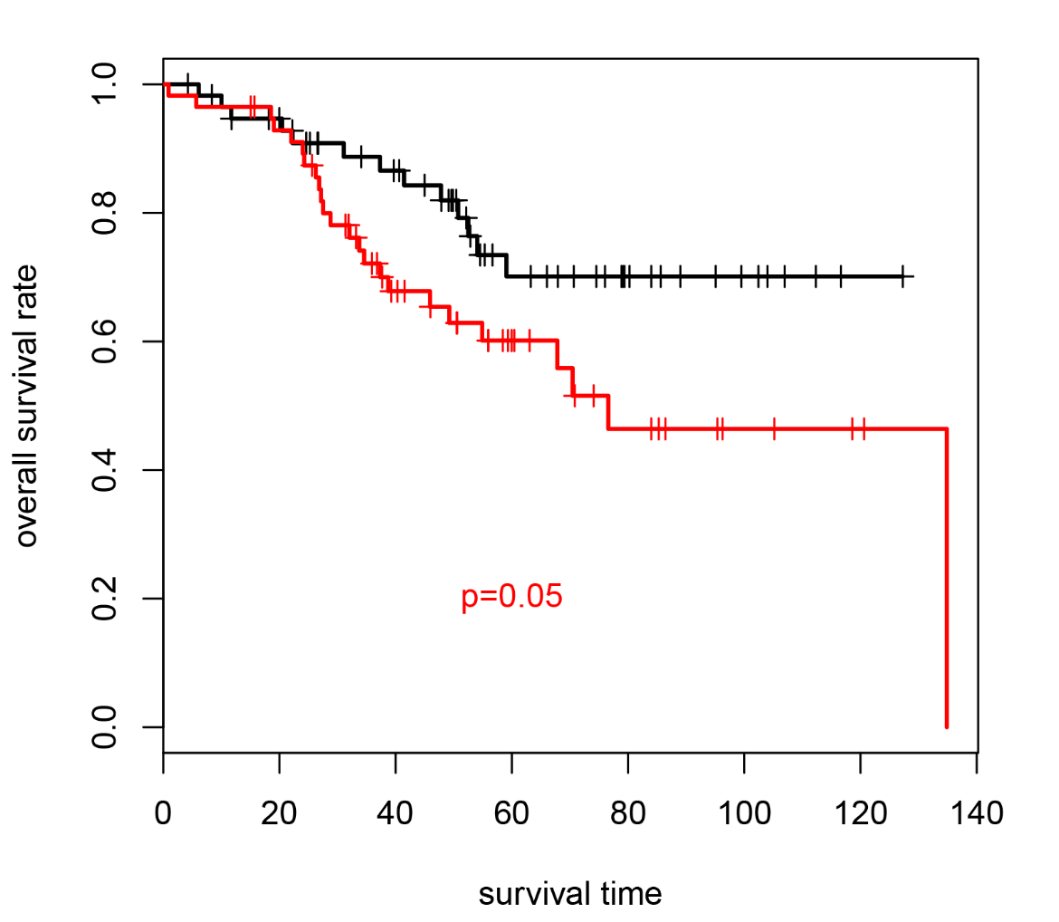

Supplement: S1 Fig — (DOCX) [file pone.0182908.s003.docx]
